# Chemical and Sensory Impacts of Accentuated Cut Edges (ACE) Grape Must Polyphenol Extraction Technique on Shiraz Wines

**DOI:** 10.3390/foods9081027

**Published:** 2020-07-31

**Authors:** Wenyu Kang, Keren A. Bindon, Xingchen Wang, Richard A. Muhlack, Paul A. Smith, Jun Niimi, Susan E. P. Bastian

**Affiliations:** 1 Waite Campus, School of Agriculture, Food & Wine, The University of Adelaide, PMB 1, Glen Osmond, SA 5064, Australia; a1642412@adelaide.edu.au (W.K.); xingchen.wang@adelaide.edu.au (X.W.); richard.muhlack@adelaide.edu.au (R.A.M.); niimi@bio.uni-frankfurt.de (J.N.); 2 The Australian Wine Research Institute, Hartley Grove, Urrbrae, Adelaide, SA 5064, Australia; keren.bindon@awri.com.au; 3 Wine Australia, Industry House, Corner Hackney and Botanic Roads, Adelaide, SA 5000, Australia; paul.smith@wineaustralia.com; 4 Institute for Molecular Biosciences, Goethe University Frankfurt, 60438 Frankfurt am Main, Germany

**Keywords:** skin fragmentation, water addition, tannin, phenolics, polysaccharides, rate-all-that-apply, astringent sub-quality, progressive profiling

## Abstract

Accentuated Cut Edges (ACE) is a recently developed grape must extraction technique, which mechanically breaks grape skins into small fragments but maintains seed integrity. This study was the first to elucidate the effect of ACE on Shiraz wine’s basic chemical composition, colour, phenolic compounds, polysaccharides and sensory profiles. A further aim was to investigate any potential influence provided by ACE on the pre-fermentation water addition to must. ACE did not visually affect Shiraz wine colour, but significantly enhanced the concentration of tannin and total phenolics. Wine polysaccharide concentration was mainly increased in response to the maceration time rather than the ACE technique. ACE appeared to increase the earthy/dusty flavour, possibly due to the different precursors released by the greater skin breakage. The pre-fermentation addition of the water diluted the wine aromas, flavours and astringency profiles. However, combining the ACE technique with water addition enhanced the wine textural quality by increasing the intensities of the crucial astringent wine quality sub-qualities, adhesive and graininess. Furthermore, insights into the chemical factors influencing the astringency sensations were provided in this study. This research indicates that wine producers may use ACE with pre-fermentation water dilution to reduce the wine alcohol level but maintain important textural components.

## 1. Introduction

Accentuated Cut Edges (ACE) is a new grape must processing technique that has recently received interest in the Australian and New Zealand wine sectors. The ACE technique, which is employed after conventional grape crushing, is a process whereby grape skins are mechanically cut into smaller fragments (6% of their original size) while maintaining seed integrity [1]. This technique provides more broken skin edges, with the goal of enhancing the extraction of phenolic components from the grape skin earlier during fermentation, while avoiding the extraction of astringent or bitter compounds potentially resulting from seed damage [2]. The development of ACE started from a northern Tasmanian vineyard in Australia working on *Vitis vinifera* cv. Pinot noir grapes [3]. The work was initiated due to the recognition that wines made from this variety can have poor colour development and low pigment stability [4]. Since a more intense colour in red wines is often associated with higher quality perception by consumers [5], the finding that ACE resulted in the intensification of wine colour was promising for the future production of higher quality wine. Compared with conventional crushing, Pinot noir wines made using the ACE technique had 50% higher wine colour density and 95% higher stable pigment concentration [1]. In addition to colour, the quality of red wine is also associated with a positive mouthfeel (or textural) properties, such as the sensation of astringency, which is known to be influenced by various phenolic components such as tannins [6]. In the study on Pinot noir, wines produced by ACE were three times higher in tannin concentration than those prepared by conventional crushing [1], and had both greater astringency and bitterness intensities [2]. Recent research demonstrated that wines with the same overall astringency intensity may possess subtle mouthfeel texture sub-quality differences e.g., velvety, puckering [7,8]. Whether ACE treatment affects these more nuanced sensations is not known. ACE-treated Pinot noir wine also had a greater intensity of fruity components, notably the aromas of banana, peach, and black currant and the flavour of dark fruit [2].

Currently, Australian wine producers are faced with managing the impacts of a shortened vintage period for many grape cultivars, termed ‘vintage compression’. This is thought to be due to the influence of climate change, with warmer growing seasons, a greater number of high temperature days and more days that have smaller diurnal temperature differences, resulting in the grapes harvested earlier and at higher sugar levels [9]. Management techniques to deal with the logistical disadvantages of processing the same tonnage of grapes in the face of vintage compression has increasingly gained importance within the wine industry [10]. In 2017, ACE was studied to address vintage compression, proposing the concept of Pressed Early Accentuated Cut Edges (PEACE) [11]. Based on the preliminary findings from PEACE, a two-day maceration on skins following ACE treatment was shown to be sufficient to extract a larger proportion of anthocyanin and tannin in Pinot noir wines relative to conventional crushing (eight days on un-fragmented skins). Thus, the ACE treatment allows the ferments to be pressed off skins earlier when compared with conventional crushing techniques, thereby highlighting the potential of PEACE to economise on tank space, pump-over logistics and labour requirements under the conditions of a compressed vintage [11].

However, as highlighted previously, ACE studies have thus far focused on Pinot noir, but one of the most planted red wine grape varieties globally, including in Australia, is *Vitis vinifera* cv. Shiraz [12], giving it a higher level of economic importance. Meanwhile, as vintage compression conditions lead to grapes destined for winemaking being harvested with increased sugar levels, the alcohol concentrations of wines made in Australia and elsewhere have also risen [13]. The high level of residual sugar or alcohol concentration in wines influences the sensory perception and hence reduces the perceived wine balance, quality or consumer preference [14]. While opportunities to manipulate wine alcohol through techniques such as earlier harvests, pre-fermentation water addition and reverse osmosis have been studied, these operations might also potentially lead to reduced wine quality [15,16,17].

Shiraz is economically important, but faces the same compressed vintage challenges. There is potential for ACE to be applied to Shiraz, however, this has not been done in a highly coloured and phenolic red grape variety before. The extent to which ACE can enhance Shiraz wine properties/sensory is unknown. Thus, the aims of the present study were to investigate, in Shiraz wine production, the impact of the ACE technique on wine chemical composition, sensory attributes and in particular, astringency and its sub-qualities. In order to determine the potential improvement provided by ACE over conventional crushing, a combination of both early pressing and water addition to wines were examined. Three treatments were investigated for both ACE and conventional crushing, whereby short skin maceration (three days on skins) was compared with a longer skin maceration time (six days). Furthermore, the longer skin contact treatments (6 days) for both standard and ACE-processed grapes were also prepared with water addition (at the pre-fermentation stage to reduce must sugar to 13.5 Baumé (Bé)). All wine treatments were chemically characterised through a number of basic wine compositional parameters, most importantly colour, phenolic composition and polysaccharide concentration. In addition, the sensory characteristics of all treatments were profiled by rate-all-that-apply (RATA) using 61 untrained participants, and then astringency and astringent sub-qualities, assessed by modified progressive profiling (PP) using a trained panel.

## 2. Materials and Methods

### 2.1. Chemicals

Reagents and reference compounds (≥97% purity) used for the high-performance liquid chromatography (HPLC), methyl cellulose precipitable (MCP) tannin method, and the modified Somers assay were purchased from Sigma-Aldrich (Castle Hill, NSW, Australia). Milli-Q water (Millipore, North Ryde, NSW, Australia) was utilised for the preparation of solutions.

For the winemaking process, potassium metabisulfite (PMS) and diammonium phosphate (DAP) were purchased from Laffort Australia (Woodville North, SA, Australia), while tartaric acid (H_2_T) was purchased from Tarac Technologies (Nuriootpa, SA, Australia). Springwater (Woolworths^®^, Unley, SA, Australia) was used for the preparation of solutions for addition to wine as well as for must dilution, to avoid chlorine impact (potential formation of 2, 4, 6-trichloroanisole).

### 2.2. Vinification Protocol

Shiraz grapes were sourced from a vineyard (35°13′ S, 138°62′ E) located in the McLaren Vale region of South Australia during the 2019 vintage. The region experiences a temperate-warm Mediterranean climate, and the mean January temperature in 2019 was 31.6 °C, and the average annual rainfall from 2000 to 2019, 518.1 mm [18]. The grapes were machine harvested from 22 year-old vines holding 7.63 tonnes/hectare average yield (1 hectare is 10,000 m^2^). A total of 600 kg was subsampled from the machine-harvested grapes. A portion of the grapes (300 kg) underwent a conventional crushing technique (Miller MC250) and was named NOACE treatment. The remaining 300 kg of grapes were crushed firstly by the same conventional approach and then underwent further processing through the Della Toffola Maceration Accelerator (DTMA, Della Toffola, TV, Italy; ACE treatment; minimum grape amount required by the winery). After the homogenisation of the two musts, the initial must conditions were measured by OenoFoss Type 41-01, and were not different to one another, having the following chemical compositions; sugar 14 Bé (1 Bé = 1.8 Brix = 18 g/L fermentable sugar = 1% potential alcohol), pH 3.39, 5.2 g/L titratable acidity (TA), 1.5 g/L malic acid, and 171.2 mg/L yeast assimilable nitrogen (YAN).

**Figure 1:**
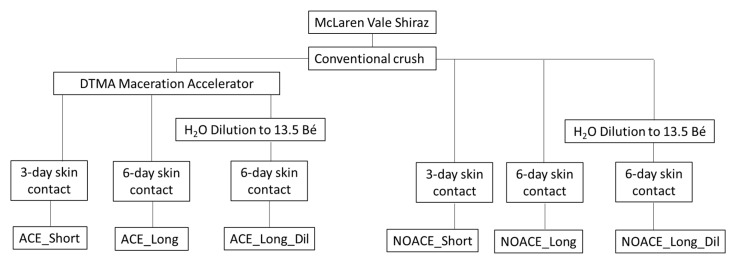
Summary of the treatments conducted on Shiraz musts prepared by the NOACE (conventional crush) or Accentuated Cut Edges (ACE) (conventional crush plus Della Toffola Maceration Accelerator (DTMA)) treatment, with six different treatments performed in triplicate.

Thereafter, both musts were acid-adjusted by the addition of 1 g/L H_2_T, 300 mg/L DAP added, followed by yeast inoculation with Enartis Ferm^®^ red fruit (batch L.ES 736051) at a rate of 200 mg/L. As shown in Figure 1, both NOACE and ACE musts were further separated (under constant stirring to maintain the ratio between the skin and juice) into 9 by 25 kg aliquots in 30 L plastic fermenters (Brewcraft, SA, Australia). Water addition treatments were conducted by the direct addition of 500 mL of spring water (i.e., no juice run off was performed) to the NOACE- and ACE-treated musts in triplicate, to reduce the sugar concentration to 13.5 Bé, based on the Australian water addition regulatory limit. The fermentation of all six treatments (Figure 1) was conducted in triplicate, in a 20 °C temperature-controlled room, with manual plunging performed twice daily (at 10 am and 4 pm, with 10 punch downs per plunging). One day after inoculation with yeast, the lactic acid bacteria were co-inoculated by the addition of VP41 (LALLEMAND^®^, Edwardstown, SA, Australia, batch 314125093016) at a rate of 1.5 mg/L. After either 3 or 6 days of skin contact, the wines were pressed at 1.5 bar for 10 min using a water bag press, transferred to 10 L glass demijohns with airlocks (Ambrosio, Italy) and stored at 18 °C until dry (the total residual sugar and malic acid of all wines were below 2 g/L and 0.4 g/L, respectively). PMS was added to achieve 60 mg/L total Sulphur, then the wines stored in a 0 °C room for one week and racked off “gross lees”. Thereafter, the wines were settled for a month at 0 °C and again racked from the “fine lees” before bottling. Wines were bottled in 375 mL dark green bottles covered with carbon dioxide and screw caps, and cellared at 16 °C for a month before being analysed.

### 2.3. Basic Wine Composition and Wine Colour Measurements

The wine samples were analysed for pH, titratable acidity (TA, as tartaric acid g/L equivalents and a TA measurement pH endpoint of 8.2), volatile acidity (VA, as g/L equivalent to acetic acid), and sulphur dioxide (SO_2_, free and total) by the Australian Wine Research Institute’s (AWRI) Commercial Services Laboratory (using the Winescan method and the method of sulphur dioxide free and total (the Thermo Fisher Discrete Analyser), respectively). The total residual sugars and malic acid levels were measured by Chemwell^®^ 2910 Automated EIA and Chemistry Analyser (Awareness Technology, Palm City, FL, USA) with the Megazyme K-FRUGL (Chicago, IL, USA) and Vintessential Enzymatic L-Malic Acid (Dromana, VIC, Australia) test kits. The alcohol level of the samples was measured with the Anton Paar Alcolyzer Wine ME and DMA 4500M (North Ryde, NSW, Australia).

The wine colour was measured by both the modified Somers assay [19] and CIELab tristimulus using the Cintra 4040, (GBC Scientific Equipment, Braeside, VIC, Australia), and the results calculated and presented as the chroma and hue angle as described previously [20].

### 2.4. Phenolic Components and Polysaccharide Analyses in Wines

Total tannin concentration for the treatments was measured by the high-throughput MCP tannin method in technical duplicates, while the total phenolic concentration was determined by the modified Somers assay in technical triplicates [19]. Furthermore, the tannins from wine samples were isolated by solid-phase extraction [21] and analysed by HPLC (Agilent 1100) following phloroglucinolysis [22] to determine the subunit composition, mean degree of polymerisation (mDP), and molecular mass (MM (phloro)) according to the conditions outlined previously [23]. All the terminal monomer subunits had their retention times authenticated using standards before measurement [23]. The tannin molecular mass was also measured by gel permeation chromatography (MM (GPC)) on an Agilent 1200 with the modifications described previously [24]. 20 mg/mL malvidin-3-glucoside in methanol was used as a standard to validate the method; this standard was removed from the freezer, equilibrated to temperature and then diluted 1:5 with *N,N*-dimethylformamide prior to analysis.

For the polysaccharide analysis, the wine samples were prepared and hydrolysed as described by Li, et al. [25], but the dialysis step was replaced by a cold, pure ethanol wash [9]. The total wine soluble polysaccharides and the monosaccharide residues following acid hydrolysis were determined by HPLC (Agilent 1100) [26]. The monosaccharides were identified and quantified using commercial standards (Sigma-Aldrich, St. Louis, MO, USA).

### 2.5. Sensory Evaluations

#### 2.5.1. Wine Descriptive Profiling by Naïve Wine Consumers Using Rate-All-That-Apply (RATA)

RATA is a rapid and flexible method that can profile different food or beverages products using naïve consumers as subjects [27,28]. For the characterisation of wines products, the discrimination and profiling abilities of RATA with naïve consumers have been validated against descriptive analysis using small, highly trained panellists [27]. Thus, a panel of 61 untrained participants (34 female and 27 male, average age 26 years) who had consumed red wine in the last 12 months assessed the Shiraz treatment wines in this study. The RATA assessment was conducted across two sessions (nine wine samples per session, all the samples from the triplicate of winemaking were assessed) under the same conditions as the work of Danner et al. [27] in computerized, individual booths with forced one minute breaks between each sample, and a five minute break after the first four wines. The participants used a seven-point intensity RATA scale (anchored from 1 = “extremely low” to 7 = “extremely high”) to evaluate 58 attributes (Table S1, definitions were provided to the consumers) across the sensory modalities of wine colour, aroma, flavour, taste, mouthfeel, and aftertaste.

#### 2.5.2. Astringency Profiles of Wines Assessed by a Trained Sensory Panel Using Modified Progressive Profiling (PP)

**Figure 2:**
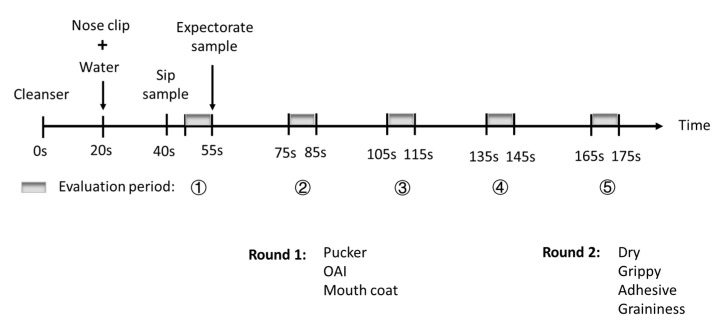
Schematic representation of the modified Progressive Profiling protocol. OAI is overall astringent intensity.

Wine astringency is a complex sensation, and is particularly hard and fatiguing for untrained individuals (normal consumers) to assess, comprehend and describe, especially the different sub-qualities of astringency perception [29]. As one study aim was to obtain an advanced understanding of the impact of ACE on the temporal perception of Shiraz wine’s texture, the astringency profiles of treatment wine samples in this study were evaluated in more detail by a trained sensory panel (*n* = 8, 3 male and 5 female, average age 51 years) using the modified PP methodology [30]. The processes of panel recruitment, training, and sample evaluation were conducted in the same manner as our previous work [30]. Seven attributes of wine astringency were evaluated as previously determined [30] including overall astringent intensity (OAI) and 6 sub-qualities (pucker, mouth coat, dry, grippy, adhesive and graininess). The intensity of attributes in each wine were rated consecutively on 15 cm scales with low and high word anchors located at 10 and 90% of the scale, respectively. The entire attribute set were assessed in two rounds, one after the other for a given wine sample. However, the PP evaluation in this study only had 5 time periods (each lasting 10 s; the first with wine in the mouth and then 4 after expectoration, with 20 s gaps between each time period). This was because the panel training had revealed that the astringency sensation had disappeared by the fifth evaluation time period (Figure 2). All wines were presented to panellists in coded black glasses, monadically in randomised order and evaluated in computerised, individual booths. The 18 wines (6 treatments × 3 replicate of winemaking) were evaluated in duplicate across four sessions (two hours, twice weekly commencing at 10 a.m. at the University of Adelaide’s Waite campus sensory facility).

### 2.6. Data Analyses

The chemical measures were analysed by one-way analysis of variance (ANOVA) at an alpha level (α) of 5% with Fisher’s least significant difference post hoc test (LSD) in XLSTAT (ver. 2016; Addinsoft SARL, Paris, France). The data from RATA were analysed by a multivariate ANOVA (at α 10%), with two-way interaction (treatment and replicate of winemaking as fixed factors, and assessor as random factor) using XLSTAT. Significantly different RATA attributes (means) were further analysed with Principal components analysis (PCA). In terms of PP assessment, the data were firstly analysed by univariate ANOVA (at α 5%) for each attribute at every single time period, with a treatment, replicate of winemaking and a replicate of sensory evaluation as fixed factors, and an assessor as a random factor using XLSTAT. Significantly different PP attributes were further analysed by the mixed assessor model canonical variate analysis (MAM-CVA) in RStudio (R ver. 3.5.1, Boston, MA, USA) with the software package CVAS (Version 1.0, written by Caroline Peltier on 3 November 2014). A partial least squares regression (PLS-R) between the significantly different attributes in PP (Y, the variables being predicted) and the significantly different chemical parameters (X, the predictor variables) were performed (stop conditions was automatic, cross-validation method used was Jackknife (LOO), and a confidence interval was 95%) using XLSTAT.

## 3. Results and Discussion

### 3.1. Basic Wine Chemical Composition and Colour

The basic wine composition resulting from the winemaking treatments is shown in Table 1. The treatments were significantly differentiated on the chemical parameters of alcohol and acid. The alcohol concentration in the water addition treatments was significantly lower in both the ACE and NOACE groups, as expected. A trend of higher pH and lower TA was found in the water addition treatments. pH is known to influence both wine colour [31] and astringency sensation [32]. The range of difference in pH was less than 0.1, which would only influence the wine colour and astringency perception negligibly, if at all.

**Figure 3:**
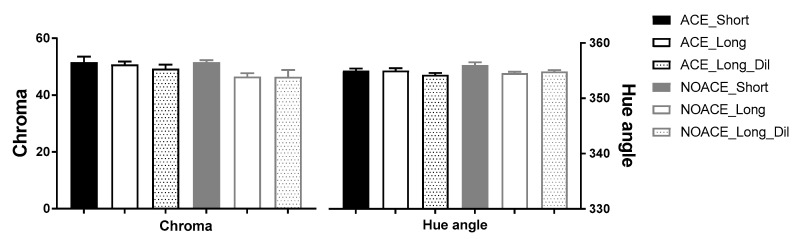
The colour of wines measured by CIELab. Shiraz wines were prepared following NOACE and ACE maceration with either 3 days (Short) or 6 days (Long) on skins, or 6 days on skins with pre-fermentation water dilution to 13.5 Bé (Long_Dil). Results are presented as the chroma and hue angle (mean ± standard deviation of triplicate fermentations).

The effect of the ACE treatment on wine colour was firstly examined using the modified Somers method (Table 2). Similar to the previous findings from Pinot noir wines [1], the ACE treatments with longer time on skins had significantly increased wine colour density and stable pigment concentration (SO_2_ resistant pigments). However, the colour enhancement in the Shiraz wines was not as large in magnitude (approx. 17% higher wine colour density and 8% higher stable pigment concentration) relative to that observed in Pinot noir (50% higher wine colour density and 95% higher stable pigment concentration). The difference in the grape varieties may have been the cause, since the Shiraz grapes have inherently more red pigments and a darker colour than Pinot noir, and compounds which may also be more readily extractable [4]. Additionally, the colour effects were measured by CIELab (Figure 3), which is expected to better approximate the colour perceived by the human eye (the modified Somers assay was based on the measurement of spectrophotometric data for several wavelengths rather than the CIELab (a whole range, 375 to 780 nm)) [33]. The chroma, represents the intensity/depth of the wine colour, and the hue angle is identified as orange, yellow, beige, brown, pink or any of the other colours. There was no significant difference in the wine colour by CIELab across the six treatments, with the chroma of all wines being approximately 50 and the hue angle 355 (i.e., a purple to red hue). The results differed to those obtained by the Somers assay and the previous Pinot noir study. The modified Somers assay was more sensitive, however, in the case of this study, the colour differences were most likely not perceivable based on the CIELab measurements.

### 3.2. Wine Total Phenolics and Total Tannin

#### 3.2.1. ACE Effects

**Figure 4:**
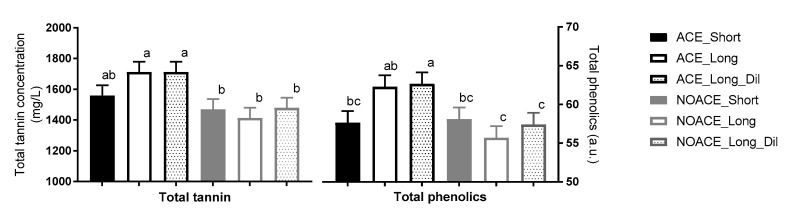
Mean total tannin concentrations and total phenolics (± standard error) of Shiraz wines prepared following NOACE and ACE maceration with either 3 days (Short) or 6 days (Long) on skins, or 6 days on skins with pre-fermentation water dilution to 13.5 Bé (Long_Dil). Different superscript letters above the bars indicate significant differences (*p* < 0.05) between treatments analysed by LSD. The a.u. in the right axis is absorbance units.

Wine phenolic components are important to wine colour, stability and quality, and they are considered to be primarily responsible for the sensation of astringency in wine [6,34,35]. As shown in Figure 4, with the exception of ACE_Short treatment, the total tannin concentrations in ACE-treated Shiraz wines were significantly higher than all NOACE treatments (F = 3.72, *p* = 0.036). Since no oak treatment was applied in this study, the tannins in wines were condensed tannins derived from the grape berries, and are located in the skin hypodermal layers, pulp, and the soft parenchyma of the seed between the cuticle and the hard seed coat [36,37]. Consistent with the previous literature [1,2], more broken skin edges provided by the ACE technique resulted in an increase in tannin extraction in Shiraz wines.

#### 3.2.2. Maceration Time and Dilution Impacts

However, the enhancement of tannin extraction in the shorter skin maceration treatment was more obvious in Pinot noir wines [11] compared to the Shiraz in this study. This might due to the DTMA machine being less destructive when cutting Shiraz skins than the original ACE equipment and Pinot noir must processing. In addition, there was no significant difference in the tannin concentration between the long skin maceration and long skin maceration plus dilution treatments. This meant that the small amount of water addition before fermentation could reduce the alcohol level without significantly influencing the total tannin concentration in Shiraz wines. This is contrary to the losses in tannin concentration observed following the dilution of Shiraz in other studies [12,17], nevertheless, the amount of water addition in the current study was much lower. The total phenolics measurement indicated that the combined estimate of wine tannins together with other phenolic components, such as non-polymeric flavonoids and derived pigments, tracked similarly to the tannin concentration (F = 3.59, *p* = 0.041). The contents of total anthocyanins were not significantly different across the six treatments, thus the differences observed in total phenolics might mainly be caused by the proanthocyanidins and polymeric pigments (SO_2_ resistant pigments).

### 3.3. Wine Tannin Composition

#### 3.3.1. Maceration Time and Dilution Impacts

The tannin composition of the winemaking treatments was determined and are shown in Table 3. The mass conversion indicates the extent to which the isolated tannin was depolymerised to resolved constituent subunits by the phloroglucinolysis method, which also reflects the confidence for the interpretation of the measured subunit compositions as representative of all tannin in the sample. Tannin mDP, which represents the average length of tannin polymers, was found to increase in the shorter skin maceration treatments relative to the longer maceration time of 6 days, independently of the treatment at crushing. The shorter skin maceration treatments also had a higher percentage of epigallocatechin subunits, indicating a greater proportion trihydroxylated material extraction from the grapes, likely reflecting a contribution from the grape skins [36,38]. On the contrary, the wines undergoing longer maceration times, including the water addition treatments, had a higher percentage of epicatechin gallate, which mainly originates from the grape seeds [36]. This indicated that when a greater proportion of grape seed tannins was transferred into wines, that this was mainly due to the maceration time, rather than the implementation of the ACE technique. It is important to highlight from the current results that the overall molecular mass (MM) of the tannin population was determined by either the phloroglucinolysis or GPC techniques had different outcomes in the current study. The MM determination by phloroglucinolysis correlated with the mDP measure, and was found to decrease in response to the extended maceration time, in agreement with an increase in seed tannin extraction. On the other hand, the MM measurement from GPC, which more accurately determines the average size of tannins as a function of their hydrodynamic volume rather than by mDP per se, was found to increase with longer maceration times. This was expected, since seed tannins are known to have a larger absolute size, or hydrodynamic volume, independent of the polymer length [23].

#### 3.3.2. ACE Effects

The observations from the tannin profiles could potentially indicate that seed integrity was maintained by ACE, since the amount of extracted tannin increased in response to ACE, but the tannins remained compositionally similar for comparable maceration times. Should ACE have disrupted the seed integrity, a decrease in mDP and an increase in proportional epicatechin gallate would have been expected earlier during maceration. According to the GPC results, the tannins derived from the ACE_Long and ACE_Long_Dil treatments were significantly larger in size than those found in the NOACE wines, suggesting more skin tannin components. Of relevance to the current study, is that larger tannins could potentially result in greater astringency perception, and this will be addressed in greater detail in the sections to follow.

### 3.4. Wine Polysaccharide Composition

ACE treatment and dilution had little impact on the polysaccharide composition (Table 4), while the total polysaccharide concentrations increased slightly as the maceration was prolonged from 3 to 6 days. In terms of the proportional composition of the individual monosaccharide residues, recovered following the acid hydrolysis of polysaccharides, fucose residues were significantly different across the treatments, but the levels were very low. At these equivalent levels, even in water, it is difficult to perceive the sweetness of fucose [39]. Significant differences across the treatments were also detected for rhamnose residues and the residues of galactose and arabinose, which are usually attributed to the rhamnogalacturonans (RGs) (e.g., RGII) and polysaccharides rich in arabinose and galactose (PRAGs), respectively; where both classes of polysaccharides are grape-derived. RG II and PRAGs have been shown to be important contributors to the mouthfeel of red wines and are thought to be negatively associated with bitterness and astringency [40,41]. Polysaccharides can also directly contribute to the mouthfeel properties of wines, such as the enhancement of the perception of palate fullness [41,42]. In addition, mannose (expected to be released by yeast as mannoproteins during fermentation and aging) was not significantly different across treatments.

### 3.5. Sensory Characteristics

#### 3.5.1. Wine Descriptive Profiling by RATA

**Figure 5:**
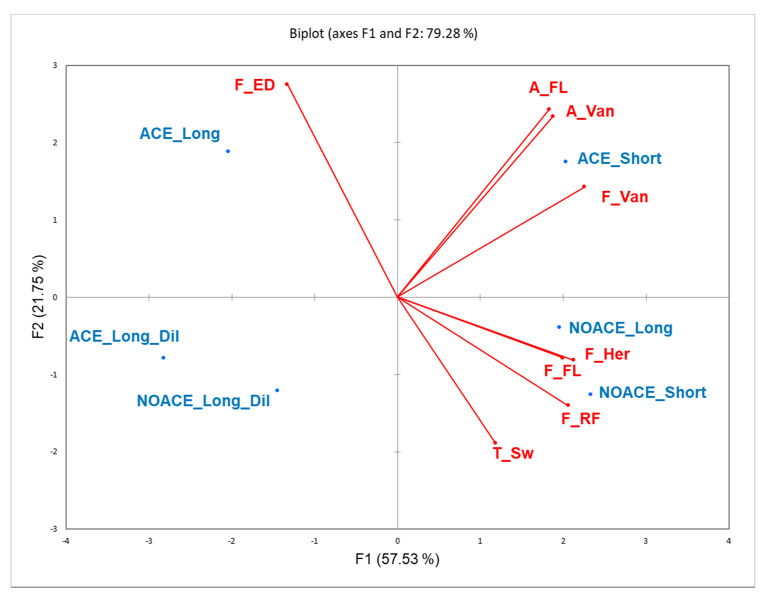
The PCA of six treatments for all the significantly different sensory attributes (*p* < 0.1) from RATA. Shiraz wines prepared following the NOACE and the ACE maceration with either 3 days (Short) or 6 days (Long) on skins, or 6 days on skins with pre-fermentation water dilution to 13.5 Bé (Long_Dil). A_FL and A_Van represent the aroma of floral/perfume/musk and the aroma of vanilla, respectively. T_Sw is the taste of sweetness. In terms of flavours, “RF”, “ED”, “FL”, “Her” and “Van” represent red fruits, earthy/dusty, floral/perfume/musk, herbaceous, and vanilla, respectively.

In the current study, the ACE technique was also studied to determine the outcomes of the sensory profile of Shiraz wines. Samples were firstly evaluated by 61 untrained wine consumers to evaluate the properties of wine colour, aroma, flavour, taste, mouthfeel, and aftertaste. Among the 58 attributes of RATA, eight were significantly discriminated between the wine treatments (*p* < 0.1) by the wine consumers, and are displayed in Table 5. The eight significantly different attributes were further visualized in a PCA plot with the sample loadings (Figure 5). Encouragingly, the winemaking triplicates for each treatment appeared consistent, as a significant treatment × replicate of winemaking sensory difference was not detected. As shown, the average intensities of vanilla were higher in the ACE treatment with short maceration on both the nose and palate. The NOACE maceration with 3 and 6 days on skins had more intense “FL” (floral/perfume/musk) flavour, but the aroma of “FL” was more intense in the ACE_Short treatment wines. These observations are interesting and the profiles of volatile aromatic compounds from wines measured by head space gas chromatography (GC) with mass spectrometry would be useful to examine this further (e.g., Monoterpenoids and C13-Norisoprenoids) [43]. The intensity of sweetness in NOACE (Short and Long) wines were higher than ACE wines, but all the samples in the current study were technically dry wines (total residual sugar were all below 2 g/L). The different perceptions in sweetness were likely to be due to the different matrix effect (such as wine alcohol level and/or possibly release of yeast-derived compounds) across the treatments [44,45]. In addition, the intensity of F_ED (flavour of earthy/dusty) in the ACE treatments were higher than in the NOACE one, especially the sample of ACE_Long. A longer skin contact combined with an increase in skin breakage could account for this observation, whereby more related flavour substances (such as 3-Isopropyl-2-Methoxypyrazine) may have been released into wine [46]. In other words, the ACE technique may have accelerated the release of certain volatile substances or their precursors. However, the intensities of the flavour of herbaceous and red fruits were negatively impacted by the ACE technique, which would be worth analysing in the future by GC, such as detecting 3-sec-Butyl-2-methoxypyrazine and Nerol for the herbaceous character, as well as β-Ionone and Furaneol for the red fruits character [43]. Furthermore, the intensity improvement of dark fruits found in ACE-treated Pinot noir wines [2] was not detected in the current Shiraz wines, which might be caused by the difference in grape varieties. Wines made by Shiraz grapes are commonly associated by the sommeliers with attributes of dark fruits [47], thus, the ACE technique did not significantly affect this character. Last, but not the least, the negative dilution effects (intensity reduction in every significantly different RATA attribute) for the pre-fermentation water addition were clear to see in the RATA evaluation, which was consistent with previous literature [12].

Wine colour differences were not detected by the consumers, in agreement with the results of the CIELab measurements taken in this study. The differences found in the chemical parameters of the acid and phenolics were not sufficiently large to elicit a sensory perception difference in the current study for either acidity or bitterness. Sixty-one untrained wine consumers did not detect a significant difference in astringency intensity between the six winemaking treatments. However, it is relevant to note that the intensity of astringency alone is insufficient to fully characterize the perception of wine astringency, that is, some wines may have a similar astringency intensity but diverse sub-qualities (textures) [48]. Meanwhile, it is also important to recognise that astringency perception is dynamic [49], in that the progression of both the intensity and sub-qualities varies depending upon the wine matrix [30,50,51,52]. Hence, a comprehensive astringency profile of each winemaking treatment was further evaluated by a trained sensory panel using PP.

#### 3.5.2. Astringency Profiles of Wines Assessed by PP

**Figure 6:**
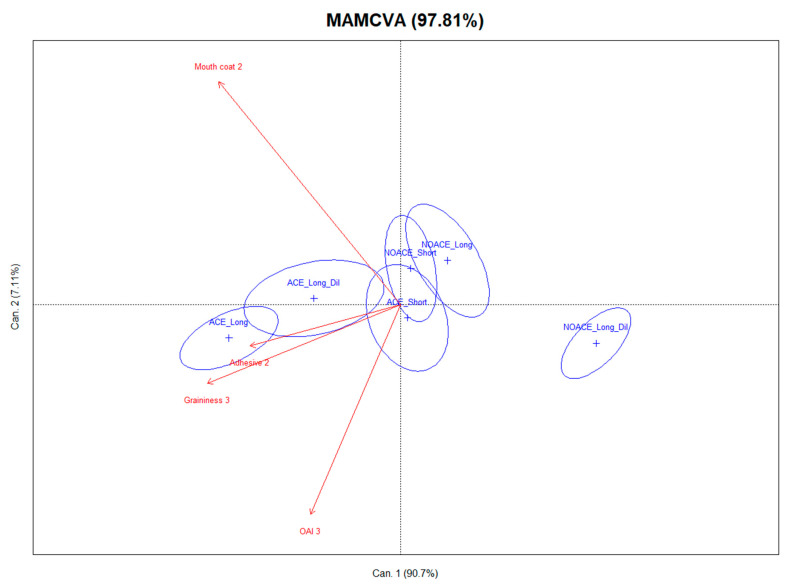
The mixed assessor model canonical variate analysis (MAM-CVA) of six treatments for all significantly different sensory attributes (*p* < 0.05) from five evaluation periods. Shiraz wines prepared following the NOACE and ACE maceration with either 3 days (Short) or 6 days (Long) on skins, or 6 days on skins with pre-fermentation water dilution to 13.5 Bé (Long_Dil). Hotelling Lawley stat = 4.458, F = 6.711 (*p* ≤ 0.001). Ellipses indicate the confidence intervals of 90%.

The PP technique is a sensory tool for the dynamic and quantitative measurements of astringency intensity and sub-qualities [30,53]. An examination of the PP panel data in this study showed good repeatability performance by the panel, as there were no significant differences between the replicates of sensory evaluation for each attribute at five evaluation periods. Significantly different astringency profiles of the Shiraz wine treatments were perceived by the panel according to the statistical analysis of the perception data (*p* < 0.05). Six treatments significantly differed by intensity for mouth coat (F = 70.27, *p* < 0.0001) and adhesive (F = 21.34, *p* < 0.0001) at the second evaluation period (20–30 s after expectoration of wine sample). Meanwhile, the intensities of OAI (F = 27.03, *p* < 0.0001) and graininess (F = 85.67, *p* < 0.0001) were significantly different by treatment at the third evaluation period (50–60 s after expectorating wine). Although a significant influence of the assessor is common in sensory evaluation (*p* < 0.0001 in the current PP), a MAM-CVA was used to reduce the scaling effect caused by the different assessors [54]. For the ease of interpretation, a joint presentation of the wine sample loadings (six wine treatments configuration plot) and the four significantly different PP attributes (sensory attribute configuration plot) are presented in one MAM-CVA plot (Figure 6). The first two canonical variates (CVs) accounted for 97.8% of the total variance ratio. As seen in Figure 6, the first CV was primarily related to the lower graininess and adhesive mouthfeel. The second CV is dominated by a mouth-coating mouthfeel, and to a lesser extent, the overall astringency intensity in the negative direction. The first axis strongly separated the less adhesive and grainy NOACE_Long dilution wines from all the other treatments. As illustrated by the lack of overlap between the confidence intervals, the grainier and more adhesive ACE_Long and ACE_Long dilution wines were clearly different from all NOACE wines. The astringency profiles of ACE_Short, NOACE_Short and NOACE_Long were similar, and clearly indicated that the astringency profiles of Shiraz wine made by conventional crushing were significantly influenced by the pre-fermentative implementation of water. Nevertheless, the use of the ACE technique not only reduced the impact of water addition on the astringency sensation, but also introduced obvious increases in the intensities of the adhesive and graininess textural sub-qualities. The enhancement provided by the ACE technique on the astringency profiles of Shiraz was consistent with what was found for Pinot noir (Sparrow, Holt, et al., 2016). As mentioned in the introduction, the most planted red wine grape variety in Australia is Shiraz, but this noble grape variety is also important for the wine industry globally (as it is grown and produces Shiraz or Syrah wines from for e.g., France, Portugal, Italy, Spain, South Africa, USA and New Zealand). This study confirmed others in Pinot noir revealing that ACE significantly enhanced the concentration of tannin and total phenolics. It also extended these findings in Shiraz wines showing that ACE was able to modify the astringency reduction caused by water dilution through an increased perception of adhesive and graininess sub-quality intensities. These positive influences on the textural quality of Shiraz wines could lead to a more extensive future application of ACE in the wine industry, not only for this but other red wine grape varieties in multiple wine producing countries.

**Figure 7:**
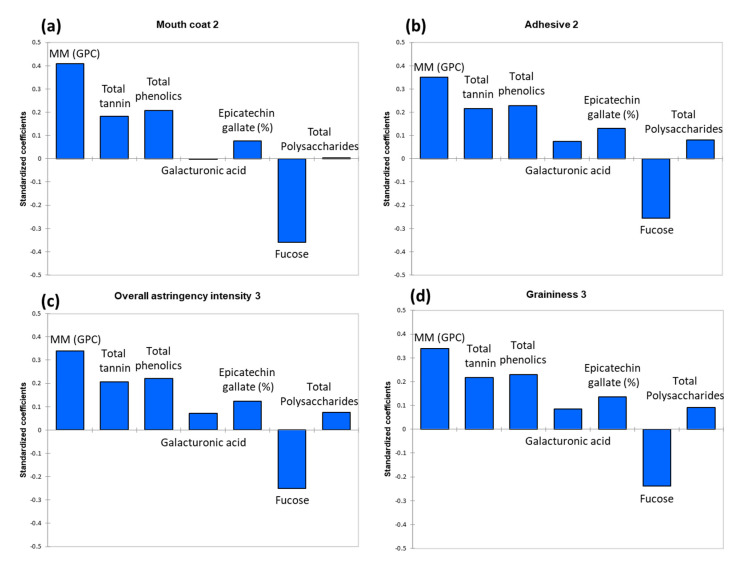
Standardised coefficients of the partial least squares regressions between the significantly different sensory attributes in PP (Y) and the selected (based on the variable importance in the projection) significantly different chemical parameters (X). MM (GPC) is tannin molecular mass determined by gel permeation chromatography at 50% elution. (**a**) Mouth coat 2 (20–30 s after the expectoration of wine sample) (**b**) Adhesive 2 (**c**) Overall astringency intensity 3 (50–60 s after expectorating wine) (**d**) Graininess 3.

To explore the underlying relationships between the significantly different mean PP sensory and wine chemistry data, the correlations between the discriminated astringency attributes (Y) and significantly different chemical parameters (X) were analysed by PLS-R (details of the first run of the PLS-R model are shown in Table S2 and Figure S1). The model was refined by a re-run of PLS-R using variables that produced variable importance in the projection (VIP) values greater than or close to 1 [55]. In the new model, the optimum number of components/latent variables required was 2, and the cross-validation index Q^2^ for two components was 0.829 (and being greater than 0.5 represented the large predictive relevance of the model) [56]. This improved model (Table 6) explained 90.8% of the variation in wine chemical composition (X-variables) and 93.5% of the variation in sensory attributes (Y-variables). To extract the relevant features for the corresponding responses (mouth coat 2, adhesive 2, OAI 3, and graininess 3), the standardized regression coefficients of the selected chemical parameters (MM (GPC), total tannin, total phenolics, galacturonic acid, epicatechin gallate (%), fucose, and total polysaccharides) are displayed, respectively, in Figure 7. The intensity of the overall astringency and three discriminated sub-qualities were all significantly (standardized coefficients were all greater than 0.3) and positively associated with tannin MM (GPC), which confirmed earlier research [30,35]. The total tannin and total phenolics concentrations and the percentage of tannin galloylation also positively contributed to OAI, which was consistent with earlier findings [30,57]. However, this was the first reported observation of the contribution of these three phenolic parameters for astringency sub-qualities. On the contrary, the negative associations of OAI and three sub-qualities with fucose residues (recovered following the acid hydrolysis of polysaccharides) were found. It is difficult to perceive the sweetness of fucose at a low level which was mentioned in Section 3.3 [39], and therefore suppress the perception of astringency [58]. However, the negative associations might support the idea that the fucose in wines co-operated with other wine matrix components, eliciting a dampening of astringency perception, and this too warrants further investigation. The chemical parameters of galacturonic acid and total polysaccharides did not predominantly influence any astringency sensation, as standardised regression coefficients were <0.1 [59]. However, it should not be neglected that phenolic and polysaccharide composition in wine did not affect astringency perception alone in many cases, and their interactions are also important [25,40,41], but this needs to be meticulously investigated in the future.

## 4. Conclusions

A new grape must processing technique (ACE) was applied for the first time on Shiraz wines to elucidate the impacts on non-volatile wine chemical compositions and sensory profiles. The ACE technique did not influence the visual colour perception of Shiraz wines, but significantly increased the concentrations of total tannin and phenolics. The polysaccharide concentration in Shiraz wines was mainly influenced by the maceration time rather than ACE technique. In addition, the greater contribution of broken skin edges provided by ACE could accelerate the release of substances related to the flavour of earthy/dusty characters into wine, which should be studied further. The pre-fermentation addition of water had significant dilution effects on the consumer-perceived wine aromas and flavours. Water addition did not reduce the concentrations of tannin or phenolics significantly, but influenced the astringency profile evaluated by a trained panel. However, the ACE technique was able to moderate the perceived astringency reduction caused by dilution through an increased intensity of perception of adhesive and graininess sub-qualities. The differences on astringency sensation could be perceived by the trained panel but not in the consumers’ assessment, however, more involved consumers may perceive these subtle changes. The knowledge generated by this study suggests that wine producers could utilise the ACE processing technique, in particular when winemakers need to modify the wine alcohol level by using pre-fermentative water dilution, and minimising the loss of important wine textural attributes. Insights into the compositional factors affecting the astringency sensation (overall intensity and sub-qualities) were provided in this study.

## Figures and Tables

**Table 1:** Basic chemical composition of the Shiraz wines prepared following NOACE and ACE maceration with either 3 days (Short) or 6 days (Long) on skins, or 6 days on skins with pre-fermentation water dilution to 13.5 Bé (Long_Dil).

	Alcohol (% v/v)	Total Residual Sugar (g/L)	pH	TA (g/L)	VA(g/L)	Malic Acid (g/L)	Free SO_2_ (mg/L)	Total SO_2_ (mg/L)
ACE_Short	^§^ 14.83 ± 0.05 a	1.63 ± 0.12	3.54 ± 0.02 ab	6.83 ± 0.06 bc	0.72 ± 0.06	<0.40	29.67 ± 0.57	51.33 ± 2.52
ACE_Long	14.57 ± 0.15 b	1.57 ± 0.21	3.53 ± 0.02 b	6.77 ± 0.06 c	0.70 ± 0.06	<0.40	30.00 ± 1.00	48.33 ± 1.52
ACE_Long_Dil	14.23 ± 0.25 c	1.37 ± 0.12	3.57 ± 0.02 a	6.67 ± 0.21 c	0.70 ± 0.21	<0.40	28.67 ± 1.52	50.33 ± 0.57
NOACE_Short	14.83 ± 0.05 a	1.93 ± 0.15	3.48 ± 0.01 c	7.17 ± 0.06 a	0.73 ± 0.06	<0.40	27.33 ± 1.52	48.67 ± 1.53
NOACE_Long	14.93 ± 0.05 a	1.33 ± 0.23	3.52 ± 0.01 b	6.97 ± 0.06 b	0.71 ± 0.06	<0.40	29.00 ± 2.00	48.00 ± 0.00
NOACE_Long_Dil	14.50 ± 0.00 b	1.47 ± 0.25	3.57 ± 0.01 a	6.70 ± 0.06 c	0.70 ± 0.10	<0.40	31.00 ± 1.00	50.00 ± 1.73
F	8.452	2.914	8.502	6.488	1.492	N/A	1.594	1.827
*p*	^†^ **0.002**	0.061	**0.002**	**0.004**	0.273	N/A	0.243	0.187

^§^ Data are the means (± standard deviation) of triplicate fermentations, analysed with one-way analysis of variance at an alpha level of 5% and Fisher’s least significant difference test. ^†^ Bold *p* values represent significant differences between treatments. A post-hoc test was run across wines within each column; values followed by the same letter in a column are not significantly different.

**Table 2:** Colour measurements by the modified Somers assay of Shiraz wines prepared following the NOACE and ACE maceration with either 3 days (Short) or 6 days (Long) on skins, or 6 days on skins with pre-fermentation water dilution to 13.5 Bé (Long_Dil).

	^§^ Wine Color Density (a.u.)	Hue	Total Anthocyanins (mg/L)	SO_2_ Resistant Pigments (a.u.)
ACE_Short	^†^ 14.74 ± 0.73 a	0.56 ± 0.00	590 ± 28	2.70 ± 0.10 ab
ACE_Long	14.76 ± 0.31 a	0.56 ± 0.01	607 ± 10	2.68 ± 0.08 ab
ACE_Long_Dil	13.75 ± 0.53 ab	0.56 ± 0.01	608 ± 20	2.56 ± 0.09 bc
NOACE_Short	14.28 ± 0.61 a	0.56 ± 0.00	587 ± 13	2.72 ± 0.06 a
NOACE_Long	12.58 ± 0.43 c	0.58 ± 0.01	557 ± 21	2.48 ± 0.06 c
NOACE_Long_Dil	12.93 ± 0.80 bc	0.58 ± 0.01	573 ± 31	2.45 ± 0.06 c
F	7.261	2.860	2.391	6.225
*p*	^‡^ **0.002**	0.063	0.100	**0.005**

^§^ Superscript represents that a.u. is the absorbance units. ^†^ Data are the means (± standard deviation) of triplicate fermentations, analysed with one-way analysis of variance at an alpha level of 5% and Fisher’s least significant difference test. ^‡^ Bold *p* values represent the significant differences between the treatments. A post hoc test was run across the wines within each column; the values followed by the same letter in a column are not significantly different.

**Table 3:** The tannin composition of Shiraz wines prepared following the NOACE and ACE maceration with either 3 days (Short) or 6 days (Long) on skins, or 6 days on skins with pre-fermentation water dilution to 13.5 Bé (Long_Dil).

	^§^ MM (Phloro) (g/mol)	^†^ mDP	Epigallocatechin (%)	epicatechin Gallate (%)	Mass Conversion (%) of Phloroglucinolysis	^‡^ MM (GPC) (g/mol)
ACE_Short	^δ^ 2893 ± 53 a	9.58 ± 0.17 a	34.6 ± 1.0 a	4.1 ± 0.0 c	40 ± 2	1793 ± 33 b
ACE_Long	2616 ± 15 b	8.64 ± 0.06 b	32.6 ± 0.7 bc	4.9 ± 0.2 a	40 ± 1	1833 ± 32 a
ACE_Long_Dil	2691 ± 82 b	8.89 ± 0.27 b	31.8 ± 1.3 c	4.7 ± 0.2 a	38 ± 2	1811 ± 12 ab
NOACE_Short	2884 ± 46 a	9.55 ± 0.15 a	34.3 ± 0.7 ab	4.1 ± 0.1 c	38 ± 2	1778 ± 23 b
NOACE_Long	2720 ± 105 b	8.99 ± 0.34 b	31.1 ± 1.0 c	4.7 ± 0.1 a	39 ± 3	1787 ± 36 b
NOACE_Long_Dil	2583 ± 114 b	8.55 ± 0.37 b	32.0 ± 1.3 c	4.4 ± 0.0 b	39 ± 4	1673 ± 24 c
F	7.752	7.968	5.595	18.758	N/A	20.966
*p*	^Φ^ **0.003**	**0.003**	**0.010**	**<0.0001**	N/A	**<0.0001**

^§^ Tannin molecular mass determined by phloroglucinolysis. ^†^ Tannin mean degree of polymerisation determined by phloroglucinolysis. ^‡^ Tannin molecular mass determined by gel permeation chromatography at 50% elution. ^δ^ Data are the means (± standard deviation) of triplicate fermentations, analysed with one-way analysis of variance at an alpha level of 5% and Fisher’s least significant difference test. ^Φ^ Bold *p* values represent the significant differences between treatments. A post-hoc test was run across the wines within each column; the values followed by the same letter in a column are not significantly different.

**Table 4:** Concentrations (mg/L) of total polysaccharides and monosaccharide residues following acid hydrolysis. Shiraz wines prepared following the NOACE and ACE maceration with either 3 days (Short) or 6 days (Long) on skins, or 6 days on skins with pre-fermentation water dilution to 13.5 Bé (Long_Dil).

	Mannose	Rhamnose	Glucuronic Acid	Galacturonic Acid	Glucose	Galactose	Xylose	Arabinose	Fucose	Total Polysaccharides
ACE_Short	^§^ 114 ± 4	40 ± 2 b	8 ± 1 b	271 ± 12 c	32 ± 4 c	123 ± 4 bc	7 ± 1	126 ± 6 c	13 ± 1 c	736 ± 28 c
ACE_Long	116 ± 4	49 ± 3 a	10 ± 0 a	307 ± 9 a	50 ± 11 ab	135 ± 2 a	7 ± 2	153 ± 4 a	16 ± 2 ab	842 ± 26 a
ACE_Long_Dil	113 ± 1	49 ± 2 a	11 ± 1 a	293 ± 9 ab	57 ± 4 ab	130 ± 3 ab	8 ± 0	148 ± 2 ab	15 ± 0 ab	824 ± 9 ab
NOACE_Short	116 ± 5	38 ± 1 b	11 ± 1 a	251 ± 9 d	61 ± 2 a	121 ± 5 c	7 ± 1	125 ± 5 c	14 ± 1 bc	744 ± 23 c
NOACE_Long	117 ± 2	46 ± 1 a	11 ± 1 a	283 ± 6 bc	47 ± 12 b	131 ± 4 ab	6 ± 1	145 ± 3 b	16 ± 1 a	802 ± 26 ab
NOACE_Long_Dil	111 ± 9	46 ± 1 a	11 ± 1 a	281 ± 4 bc	50 ± 5 ab	129 ± 6 abc	7 ± 1	144 ± 7 b	17 ± 1 a	795 ± 35 b
F	0.602	15.325	3.947	14.743	5.155	3.898	1.029	19.492	5.621	8.146
*p*	0.700	^†^ **<0.0001**	**0.024**	<**0.0001**	**0.009**	**0.025**	0.443	**<0.0001**	**0.007**	**0.001**

^§^ Data are the means (±standard deviation) of triplicate fermentations, analysed with one-way analysis of variance at an alpha level of 5% and Fisher’s least significant difference test. ^†^ Bold *p* values represent significant differences between the treatments. A post-hoc test was run across the wines within each column; values followed by the same letter in a column are not significantly different.

**Table 5:** The significantly different attributes across the treatments from rate-all-that-apply (RATA) detected by a multivariate ANOVA (at α 10%).

Attribute	Definition	F	*p*
**Aroma**			
A_FL	Floral/perfume/musk	2.292	0.044
A_Van	Vanilla	2.112	0.062
**Taste**			
T_Sw	Sweet	2.201	0.052
**Flavour**			
F_RF	Red fruits (e.g., raspberry, strawberry, red cherry, and red current...)	2.036	0.071
F_ED	Earthy/dusty	3.210	0.007
F_FL	Floral/perfume/musk	1.999	0.076
F_Her	Herbaceous	1.984	0.079
F_Van	Vanilla	2.144	0.058

**Table 6:** Fit statistics of the partial least squares regression (two components) analysis between the discriminated astringency attributes (Y) and significantly different chemical parameters (X).

	Mouth Coat 2	Adhesive 2	Overall Astringency Intensity 3	Graininess 3
Cumulative Q^2^ quality index	0.721	0.918	0.757	0.924
R^2^	0.897	0.971	0.899	0.974
Std. deviation	0.913	0.549	0.964	0.209
Mean-square error	0.334	0.120	0.372	0.017
Root mean squared error of prediction	0.578	0.347	0.610	0.132

Mouth Coat 2 and Adhesive 2 attributes were perceived 20–30 s after the expectoration of the wine samples. Overall Astringency Intensity 3 and Graininess 3 attributes were evaluated 50–60 s after the expectoration of the wine samples.

## Supplementary Materials

The following are available online at https://www.mdpi.com/2304-8158/9/8/1027/s1, Table S1: List of sensory attributes scored in the rate-all-that-apply (RATA) assessment, Table S2: The model performance for the first run of partial least squares regression, and Figure S1: The plot of the first run of Partial Least Squares regression between the significantly different sensory attributes from PP (in blue) and significantly different chemical parameters (in red).
