# Supplementary material for: Chemical and Sensory Impacts of Accentuated Cut Edges (ACE) Grape Must Polyphenol Extraction Technique on Shiraz Wines
Source: Foods. 2020 Jul 31;9(8):1027. doi: 10.3390/foods9081027 (PMC7466200; doi:10.3390/foods9081027)
Supplement: Supplementary file 1 [file foods-09-01027-s001.pdf]

Supplementary Materials：

Table S1. List of sensory attributes scored in the rate-all-that-apply (RATA) assessment.

	No.	Attribute abbreviation		No.	Attribute abbreviation	Definition
Color	1	C_R				Intensity of red color
	2	C_P				Intensity of purple color
	3	C_B				Intensity of brown color
	No.	Attribute abbreviation		No.	Attribute abbreviation	Definition
Aroma	4	A_DF	Flavor	26	F_DF	Dark fruit (e.g. blackberry, blackcurrant, plum, and dark cherry...)
	5	A_RF		27	F_RF	Red fruit (e.g. raspberry, strawberry, red cherry, and red current...)
	6	A_DrF		28	F_DrF	Dried fruit (e.g. prune, raisins, fig and dried apricote...)
	7	A_Ja		29	F_Ja	Jammy
	8	A_Con		30	F_Con	Confectionery (e.g. candy, lolly, fruit drops...)
	9	A_Choc		31	F_Cho	Chocolate
	10	A_CN		32	F_Co	Coconut
	11	A_CV		33	F_CV	Cooked vegetables (e.g. cabbage and beans...)
	12	A_ED		34	F_ED	Earthy / Dusty
	13	A_EM		35	F_EM	Eucalypt / Mint
	14	A_FL		36	F_FL	Floral / Perfume / Musk
	15	A_FFM		37	F_FFM	Forest floor / Mushrooms
	16	A_GP		38	F_GP	Green pepper / Capsicum
	17	A_Her		39	F_Her	Herbaceous
	18	A_Le		40	F_Le	Leather
	19	A_Pep		41	F_Pep	Pepper (black and white pepper)
	20	A_Sav		42	F_Sav	Savoury / Meaty / Gamey
	21	A_Sp		43	F_Sp	Spice (e.g. anise, clove, cinnamon, liquorice, and nutmeg...)
	22	A_SS		44	F_SS	Stemmy / Stalky
	23	A_TS		45	F_TS	Toasty / Smoky
	24	A_Van		46	F_Van	Vanilla
	25	A_Wo		47	F_Wo	Woody (e.g. cedar, pencil shavings, and cigar box...)
Taste	48	T_B				Bitterness
	27	T_Sw				Sweet
	28	T_A				Sour / Acidity
Mouthfeel	51	MF_B				Wine body
	52	MF_OH				Alcohol level / Heat
	53	MF_Ast				Astringency / Tannin
	54	MF_Sm				Smoothness
	55	MF_Ro				Roughness
	56	MF_Vis				Viscosity (the resistance of the wine when you move it around on the palate)
Aftertaste	57	AT_F				Length of the aftertaste of fruit flavors
	58	AT_NF				Length of the aftertaste of non-fruit flavors

Table S2. The model performance for the first run of Partial Least Squares regression. The discriminated astringency attributes are Y-variables and significantly different chemical parameters are X-variables.

Model quality of Partial Least Squares regression		
Statistic	Comp1	[a]Comp2
[b]Q^2 cum	0.185	0.748
[c]R^2Y cum	0.637	0.944
[c]R^2X cum	0.417	0.750

Variable Importance in the Projection (VIP):		
Variable	VIP for Comp1	VIP for Comp2
GPC MM (g/mol)	2.073	**1.849**
Total tannin	1.886	**1.555**
Total phenolics	1.824	**1.502**
Galacturonic acid	1.181	**1.037**
%gall	1.179	**1.021**
Fucose	0.666	**0.995**
Total Poly	0.978	**0.928**
Galactose	0.907	0.862
Rhamnose	0.739	0.855
Arabinose	0.738	0.828
HPLC MM (g/mol)	0.031	0.799
%Tri-OH	0.283	0.796
mDP	0.012	0.787
pH	0.240	0.772
Alcohol	0.589	0.705
Titratable acid pH 8.2	0.322	0.689
Glucuronic acid	0.335	0.539
Glucose	0.148	0.172

[a] The optimum number of components/latent variables required for this model was 2 (determined by the automatic function in the XLSTAT).

[b] The Q^2 cumulative index measures the global goodness of fit and the predictive quality of the models.

[c] The cumulative R^2Y and R^2X cum that corresponds to the correlations between the explanatory (X) and dependent (Y) variables with the components are very close to 1 with 2 components.

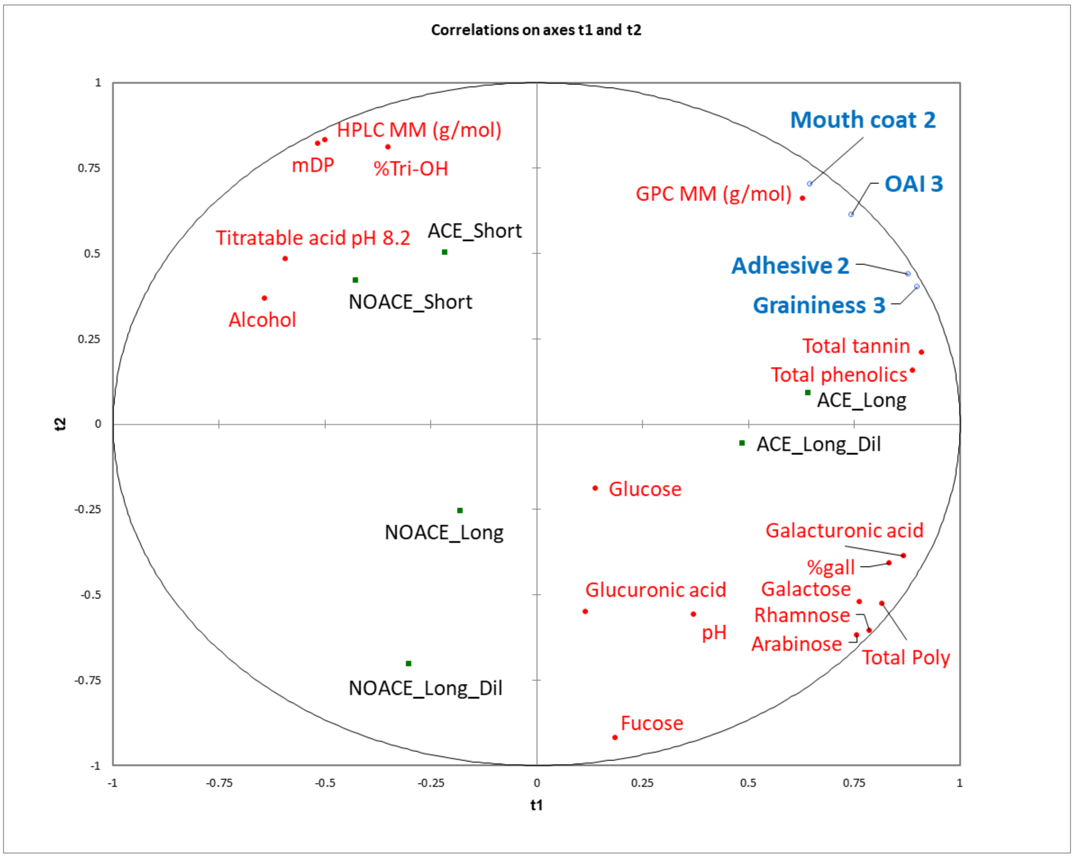

Figure S1. The plot of the first run of Partial Least Squares regression between the significantly different sensory attributes from PP (in blue) and significantly different chemical parameters (in red). Shiraz wines (in black) prepared following NOACE and ACE maceration with either 3 days (Short) or 6 days (Long) on skins, or 6 days on skins with pre-fermentation water dilution to 13.5 Bé (Long_Dil). %Tri-OH, %gall and total poly are epigallocatechin(%), epicatechin gallate (%) and total polysaccharides, respectively.
